# A novel chemotherapeutic protocol for peritoneal metastasis and inhibition of relapse in drug resistant ovarian cancer

**DOI:** 10.1002/cam4.1631

**Published:** 2018-06-21

**Authors:** Siddik Sarkar, Obeid M. Malekshah, Alireza Nomani, Niket Patel, Arash Hatefi

**Affiliations:** ^1^ Department of Pharmaceutics Rutgers, The State University of New Jersey Piscataway NJ USA; ^2^ Rutgers Cancer Institute of New Jersey New Brunswick NJ USA

**Keywords:** cancer stem cells, combination chemotherapy, ovarian cancer, peritoneal metastasis, recurrence, tumorsphere

## Abstract

The majority of ovarian cancer patients are diagnosed in late stages of the disease, in which the tumor cells have leaked into the peritoneum and are present as tumorspheres. These tumorspheres are rich in cancer stem‐like cells (CSCs), which are resistant to therapy and are a major source of relapse. The purpose of this research was to identify a safe therapeutic approach that could eradicate the peritoneal CSC‐rich tumorspheres and inhibit relapse. Highly metastatic ascitic cells (OVASC‐1) that are resistant to standard‐of‐care chemotherapy due to upregulation of MDR1 gene were obtained from a patient with ovarian carcinoma and recurrent disease. CSC‐rich tumorspheres were generated, characterized, and treated with different chemotherapeutics. The most effective drug combination that could eradicate tumorspheres at nanomolar levels despite upregulation of MDR1 gene was identified. Luciferase‐expressing OVASC‐1 cells were implanted in the peritoneum of nude mice and treated with the identified drug combination. The progression of disease, response to therapy and recurrence were studied by quantitative imaging. Toxicity to abdominal tissues was studied by histopathology. Mice implanted with intraperitoneal (IP) OVASC‐1 xenografts showed limited response to combination therapy with cisplatin/paclitaxel at the maximum tolerated dose. Despite overexpression of MDR1 on OVASC‐1 cells, mice treated with our combination IP low‐dose MMAE and SN‐38 chemotherapy showed complete response without relapse. No signs of toxicity to abdominal tissues were observed. While MMAE and SN‐38 are not administered as free drugs due to their high potency and potential for systemic toxicity, our low‐dose localized therapy approach effectively restricted the cytotoxic effects to the tumor cells in the peritoneum. Consequently, maximum efficacy with minimal adverse effects was achieved. These remarkable results with IP low‐dose combination chemotherapy encourage investigation into its potential clinical application as either first‐line therapy or in cases of acquired resistance to cisplatin and paclitaxel.

## 1 INTRODUCTION

Ovarian cancer is associated with the highest mortality rate of all gynecologic malignancies in the United States and carries an overall 5‐year survival rate of 45%. Most patients initially respond to standard treatments combining surgery and chemotherapy; however, the majority acquires multidrug resistance and succumb to their disease because of relapse.^1^ It is believed that this clinical course is in line with the cancer stem cell model.^1^, ^2^ According to the cancer stem cell theory, cancer‐initiating cells (also termed cancer stem‐like cells, or CSCs), play a major role in tumor recurrence and metastatic spread. Therefore, it is crucial to design a strategy that eradicates both differentiating ovarian cancer cells and CSCs during early‐stage treatment. The standard of care for an ovarian cancer patient in the early stages consists of debulking surgery followed by six rounds of chemotherapy with platinum‐based drugs and paclitaxel. The intent is to rid the patient of all tumors and remaining cancer cells, thereby minimizing the possibility of relapse and metastasis to distant sites. Clinical data show that after tumor debulking, addition of intraperitoneal (IP) chemotherapy to intravenous (IV) therapy significantly improves ovarian cancer outcomes in comparison to IV chemotherapy alone because ovarian cancer cells tend to leak into the abdominal fluid and remain as single or small spheroids.^3^, ^4^ If left untreated, these ascitic cells, which are rich in CSCs, continue to grow and generate new tumors in both local and distant sites. The major deficiency that currently exists is that the ovarian CSCs in the abdominal fluid are usually very resistant to chemotherapy, and in some cases high doses of chemotherapeutics are needed for their effective eradication. As a result, IP treatment is associated with significant side effects, which in such cases forces the physicians to halt the chemotherapy. To overcome this deficiency, it was our objective to develop a chemotherapeutic approach that is not toxic to normal tissues but can effectively kill the CSC‐rich tumorspheres in the abdominal fluid and inhibit relapse. As a first step toward achieving our objective, we obtained ascites‐derived malignant cells from a patient with recurrent advanced ovarian carcinoma. These ascitic cells overexpress MDR1 and show resistance to chemotherapy with paclitaxel.^5^ Using these ascitic cells, termed OVASC‐1, we first generated CSC‐rich tumorspheres in cell culture under nonadherent conditions. The tumorspheres were then exposed to various anticancer drugs to determine their sensitivity to chemotherapeutics. Using the obtained in vitro data, we created various treatment protocols and examined both the efficacy and toxicity of each approach in nude mice bearing IP xenografts. Ultimately, we identified a low‐dose chemotherapeutic approach that could not only kill the xenografted tumorspheres in the peritoneum, but also inhibit relapse. The progression of disease, response to therapy and inhibition of relapse were evaluated by quantitative live‐animal imaging. The toxicity of the optimum therapeutic protocol to normal tissues was studied by histopathology.

## 2 MATERIALS AND METHODS

### 2.1 Chemicals and reagents

Ten percent bovine serum albumin (BSA, Sigma‐Aldrich, USA) was prepared in D‐PBS (Life Technologies Corporation, NY, USA) and kept at −20°C. Recombinant human epidermal growth factor (EGF) and basic fibroblast growth factor (bFGF) (Life Technologies Corporation) were reconstituted in D‐PBS at concentrations of 200 μg/mL and 100 μg/mL, respectively. A stock solution of 10 mg/mL (16.23 mmol/L) Hoechst 33342 was prepared in deionized water and stored at −20°C. A stock solution of 1 mg/mL cisplatin (Sigma‐Aldrich) was prepared in 0.9% saline, whereas stock solutions of 5 mg/mL monomethyl auristatin E (MMAE) (MedChemExpress, NJ, USA), 1 mg/mL SN‐38 (Cayman Chemical, MI, USA), 50 mg/mL paclitaxel, 30 mg/mL etoposide, 50 mg/mL 6‐methylpurine (6‐MP), and 50 mg/mL 5‐Fluorouracil (5‐FU) (Sigma‐Aldrich) were prepared in DMSO and stored at −20°C until used.

### 2.2 Cell culture

As a precaution, all cell lines used in this study were first treated with BM‐Cyclin (Sigma) to make them mycoplasma‐free and then sent in 2016 to the University of Arizona Genetics Core, Cell Authentication Services, for authentication. The A2780 cell line (originally from Sigma) was a kind gift from the laboratory of Dr. T. Minko (Rutgers University) and maintained in RPMI‐1640 supplemented with 10% FBS. Malignant ovarian cancer cells (OVASC‐1) were originally drawn from the ascitic fluid of an ovarian cancer patient at Rutgers Cancer Institute of New Jersey, deposited into the Biorepository Center (de‐identified), and then transferred to our laboratory. OVASC‐1 cells were maintained in RPMI‐1640 supplemented with 15% FBS and 2.5 μg/mL insulin.^5^, ^6^ The media was changed every other day to maintain the health of the cells. To obtain OVASC‐1 cells with stable expression of the luciferase gene, OVASC‐1 cells were transfected with pGL4.5‐[CMV/luc2/hygro] (Promega Corporation, WI, USA). Clones were selected under continuous exposure to 400 μg/mL of hygromycin as described previously.^7^ All cell lines were cultured at 37°C in a 5% CO_2_ 95% air‐humidified incubator.

### 2.3 Generation, propagation and characterization of tumorspheres

Tumorspheres were generated by transferring 2 × 10^4^ ovarian cancer cells (OVASC‐1 and A2780) per mL of MEBM supplemented with 0.4% BSA, 20 ng/mL EGF, 10 ng/mL bFGF, 5 μg/mL insulin (Sigma‐Aldrich, MO, USA), and 1% antibiotic‐antimycotic solution into an ultra‐low attachment 6‐well plate (Sigma‐Aldrich). Cells were allowed to grow until the tumorsphere size reached >100 μm. The tumorspheres were then dissociated using Accumax (Innovative Cell Technologies, Inc. CA, USA) to make a single cell suspension and reseeded in MEBM medium with supplements to create secondary tumorspheres. This process was repeated at least three times to enrich the tumorspheres with CSCs. The live/dead status of cells inside the tumorspheres was studied by staining with calcein‐AM (green) and Hoechst 33342 (red) fluorescent dyes for 2‐3 hr at 4°C followed by observation under a fluorescence microscope.

To evaluate the expression of stem cell markers, tumorspheres were dissociated and total RNA from 10^6^ number of the ovarian cancer cells was extracted with a commercially available RNA extraction kit (Qiagen, MD, USA) as per manufacturer's instructions. Total RNA (0.5‐1 μg) was reverse‐transcribed with SuperScript II reverse transcriptase (Life Technologies) using random hexamer priming. Quantitative real‐time PCR was performed with an Applied Biosystems 7500 Real‐Time PCR system (Applied Biosystems, Life Technologies Corporation) using Taqman Universal qPCR Master Mix (Life Technologies Corporation) and respective probes as per manufacturer's instructions. Data are presented as mean ± SD (n = 4).

### 2.4 Evaluation of tumorsphere proliferation rate by PKH26 dye

Single cell suspensions of 2 × 10^6^ cells were prepared and labeled with PKH26 dye in diluent C (1:500 dilution, yielding a final concentration of 2 μmol/L) as per instructions for PKH26 Red Fluorescent Cell Linker Kits for General Cell Membrane Labeling (Sigma‐Aldrich). The cells were washed twice to remove any unbound dye and then suspended in MEBM with supplements at the density of 6 × 10^4^ cells/well in a 6‐well low‐adherent plate. Tumorspheres were collected at different time (day) intervals, centrifuged, and dissociated into a single cell suspension with Accumax/D‐PBS (1:1) prior to analysis with a Gallios flow cytometer (Beckman Coulter, Inc. CA, USA). Proliferation index (PI) and various other cell tracking parameters were studied and quantified using Modfit LT V4.1.7 software with the cell tracking wizard module.

### 2.5 Dose response curve of various chemotherapeutic drugs

Ovarian cancer cells were transferred into an ultra‐low attachment plate at the seeding density of 2 × 10^3^ cells/well in 200 μL of MEBM with supplements to generate tumorspheres as mentioned above. The tumorspheres were treated with increasing doses of MMAE (0.1‐10 nmol/L), SN‐38 (1‐100 nmol/L), etoposide (0.1‐10 μmol/L), cisplatin (1‐100 μmol/L), 6‐MP (1‐100 μmol/L), and 5‐FU (1‐100 μmol/L) 3 days post seeding. The tumorsphere number (*N*) and radius (*R*) were analyzed 14 days postseeding. Total cell content (TCC) in each well was calculated by considering the radius of one cell (*r*) and combining both *N* and *R* parameters as shown below:

$$\text{Volume}\;\text{of}\;\text{one}\;\text{cell}\;=\;\frac{4}{3}\pi r^{3}$$

$$\text{No.}\;\text{cells}\;\text{in}\;a\;\text{sphere}\;\left(1,\;R,\;r\right)\;=\;\frac{R^{3}}{r^{3}}$$

$$\text{TCC}\;\left(N,R,r\right)\;=\;N{\left(\frac{R}{r}\right)}^{3}\;\infty N\cdot R^{3}$$

### 2.6 Determination of combination index value

OVASC‐1 cells were seeded in 96‐well plates at a density of 5 × 10^3^ cells per well. Twenty‐four hours later, cells were treated with SN‐38 at concentrations ranging from 0 to 200 nmol/L and MMAE at concentrations ranging from 0 to 20 nmol/L at a 1:10 ratio. After 72 hours, the media was replenished with 100 μL of fresh media containing 10 µL WST‐1 reagent (Sigma‐Aldrich, PA, USA). After incubation for 2 hours at 37°C, the absorbance was determined with a microplate reader (Tecan, Switzerland). The absorbance of each well was normalized to the negative control (untreated cells) in order to determine the cell viability. To determine the antagonistic, additive, or synergistic effects of combination therapy, we calculated the combination index (CI) using the fractional product method.^7^, ^8^ Then, CI was plotted against the fraction of killed cells, also referred to as fraction affected (Fa).

$$\text{Fa}=1-\frac{{\text{OD}}_{\mathrm{Treatment}}}{{\text{OD}}_{\mathrm{Control}}}$$

where OD_Control_ = absorbance of control or untreated cells and OD_Treatment_ = absorbance of cells treated with drug(s).

A CI value of less than 1 indicates synergism, a CI value >1 indicates antagonism, and a CI value of 1 indicates an additive effect.

### 2.7 Evaluation of the therapeutic efficacy and inhibition of relapse in vivo

All in vivo studies described here were performed according to the guidelines of the Rutgers University Institutional Animal Care and Use Committee (Protocol# 11‐001). Outbred homozygous nude J:NU (Foxn1^nu^/Foxn1^nu^) female mice (5‐6 weeks old) were purchased from The Jackson Laboratory (Bar Harbor, ME). In vivo peritoneal xenografts were generated using OVASC‐1‐*luc* cells (OVASC‐1 cells stably expressing the luciferase gene). 5 × 10^6^ cells were resuspended in 500 μL of D‐PBS and then injected IP using a 25G needle. Stable expression of luciferase in mouse abdomen was monitored periodically for 7 days before the start of treatment on day 8. Tumorsphere bearing mice were then randomized into control and treatment groups. Unless otherwise specified, the chemotherapeutic(s) were administered IP in a total volume of 2 mL vehicle once per week for five doses. The vehicle solution was composed of 1% cremophor:ethanol (50:50) and 99% saline (0.9%).

Bioluminescence imaging (BLI) was conducted as mentioned previously for monitoring disease progression.^7^, ^9^ Xenografted nude mice were imaged weekly 1 day before the administration of drugs. Data acquisition was conducted using the IVIS Lumina III Imaging System, and images were analyzed using the Living Image 4.5 module. To study treatment response, fold change in BLI signal (ie BLI measurement at the end of treatment/BLI measurement prior to the first treatment) was calculated for each mouse. To assess recurrence, mice were imaged once every 2 weeks beginning with the final day of the treatment. Observable indicators of health (eg appetite, posture, movement) and weight were continuously monitored to detect any chemotherapeutic‐related toxicities or morbidities resulting from ascitic burden. Loss of more than 10% body weight was considered treatment‐related toxicity. When toxicity was observed, either the treatment was discontinued or mice were euthanized.

### 2.8 Histopathology study

Organs of all three mice in untreated control group and the group treated with SN‐38 1 (mg/kg) and MMAE (100 μg/kg) were collected at the end of the treatment period immediately following euthanasia. Organs were washed in 0.9% saline solution, placed inside a Cryomold (Fisher Scientific) filled with Tissue‐Plus^™^ O.C.T Compound (Fisher Scientific), and then snap frozen in liquid nitrogen. Cryosectioning was performed at the Rutgers Cancer Institute of New Jersey Histopathology Core Facility using a cryostat, followed by fixation and hematoxylin and eosin (H&E) staining. The slides were interpreted by a histopathologist at the Rutgers Robert Wood Johnson University Hospital. Photomicrography was conducted using a Leica microscope (20× objective).

## 3 RESULTS AND DISCUSSION

### 3.1 Ovarian cancer tumorspheres are enriched with CSCs

**Figure 1:**
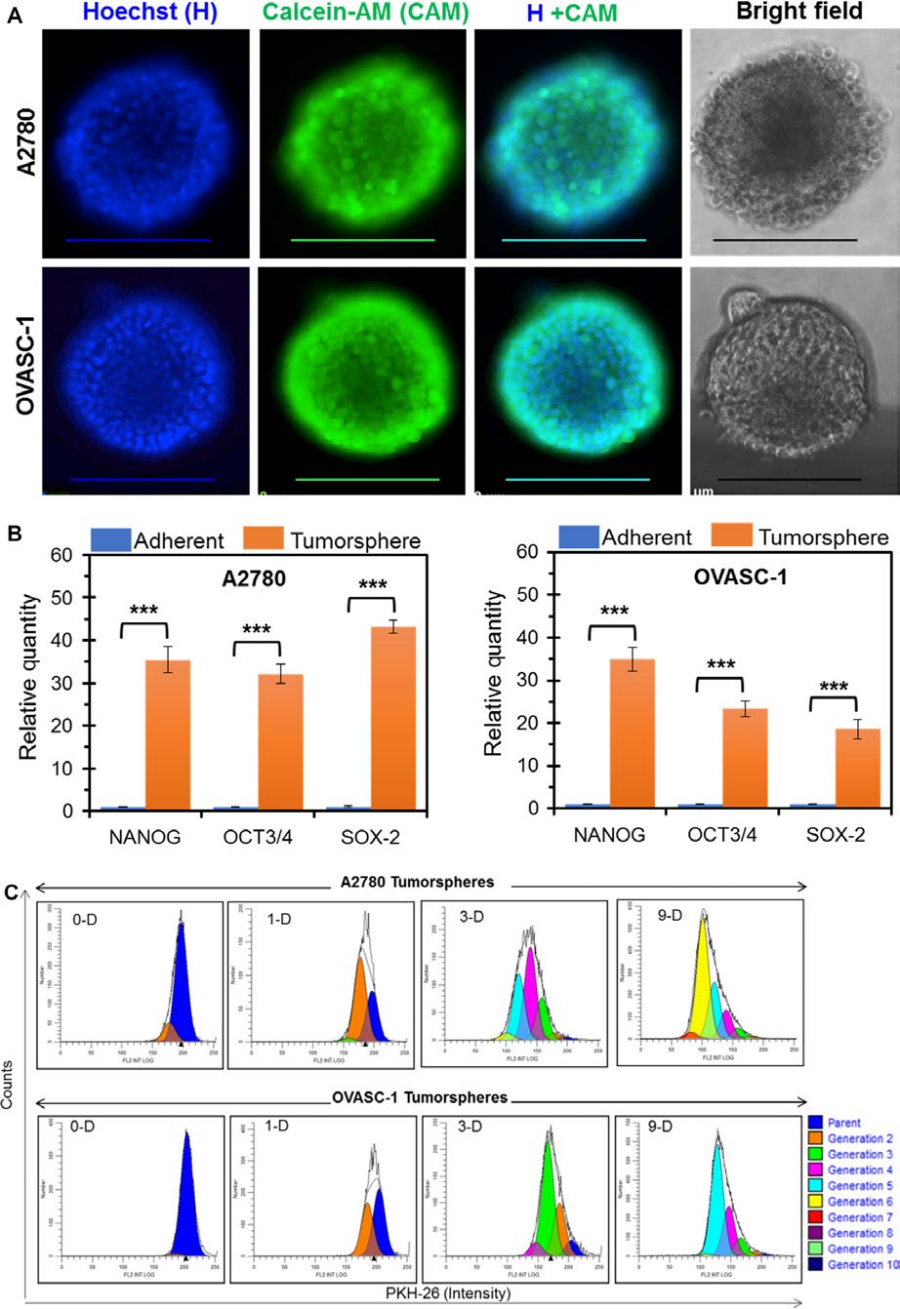
Generation and characterization of CSC‐enriched tumorspheres of A2780 and OVASC‐1 cells. A, Tumorspheres in suspension were stained with Calcein‐AM (CAM) and Hoechst 33342 (H) dyes and observed under a fluorescence microscope. Scale bar = 200 µm. B, Relative expression of stem cell markers in ovarian cancer cells under adherent and nonadherent conditions as quantified by real‐time PCR. Data are presented as mean ± SD (n = 4). ***Indicates significance (paired *t* test, *P *<* *.001). C, CSC‐rich tumorspheres showing heterogeneous population with different proliferation kinetics. Histograms of A2780 and OVASC‐1 tumorspheres from day zero (0‐D) until day nine (9‐D) postseeding, representing progenitors or daughter cells of different generations

Subpopulations of ovarian tumor cells in ascitic fluid display cancer stem‐like properties including increased resistance to therapies, the ability to spread into distant sites, and the ability to induce cancer recurrence.^10^, ^11^ Since malignant cancer cells in the ascitic fluid are a major source of mortality in ovarian cancer patients, development of a treatment protocol that can effectively eliminate these cells is of great interest. Suspended cancer cells in the form of spheroids (tumorspheres) represent a suitable in vitro experimental model of ovarian cancer ascites. Therefore, we generated ovarian cancer tumorspheres in low‐adherent culture plates using an established A2780 ovarian cancer cell line and OVASC‐1 ascitic cells obtained from the IP fluid of an ovarian cancer patient with recurrent disease. We observed that both A2780 and OVASC‐1 cells had tumorsphere forming capability even after seven passages under low‐adherent conditions (Figure 1A). To characterize and better understand the change in the CSC population before and after tumorsphere formation, we measured the expression of classical stem cell markers by real‐time PCR. In comparison to the cells cultured under adherent conditions, the A2780 cells inside the tumorspheres showed 35.4 ± 3.2, 32.2 ± 2.4, and 43.3 ± 1.5 fold positive change in mRNA expression levels of NANOG, OCT‐3/4 and SOX‐2, respectively. Similarly, significant overexpression in mRNA levels of the same genes was observed in OVASC‐1 cells (Figure 1B). The results of this study, as evidenced by the overexpression of major classical stem cell markers, show that the procedure used to generate tumorspheres significantly increases the percentage of the CSC population, resulting in CSC‐enriched tumorspheres.

To assess whether the cancer cells in the tumorspheres are dormant or have the potential to grow in size under harsh low‐adherent conditions, we characterized them further by examining their growth kinetics. For this purpose, we first stained the cells with PKH26 red tracer dye, then reseeded them under nonadherent conditions to form tumorspheres, and finally dissociated them for analysis after predetermined time intervals. The results of this experiment showed that the majority of the cells in tumorspheres were in a proliferation stage by day 3 postseeding. Notably, 1% of A2780 and 7% of OVASC‐1 cells were in the parental G1 stage (Figure 1C, and Table S1). This result indicates that the tumorspheres are heterogeneous with regard to growth kinetics. By day 9, almost all of the A2780 and OVASC‐1 tumorsphere cells were distributed in the G6 (generation 6) or G5 proliferation stage, respectively. The PI, which indicates the average number of cell divisions undergone by the proliferating cells, was calculated to be 15.06 for A2780 and 8.78 for OVASC‐1. These data show that the tumorspheres of both cell lines were actively growing under low‐adherent conditions. This is an important observation, as it could explain the potential difficulty of effectively killing such tumorspheres in ascitic fluids: the constantly growing suspended tumorspheres could generate substantial resistance to permeation by chemotherapeutics.^11^ In addition, the data indirectly suggest that the OVASC‐1 tumorspheres, which exhibit a slower growth rate, could show decreased sensitivity to antimitotic chemotherapeutics.

### 3.2 Evaluation of the tumorsphere sensitivity to drug treatment

**Figure 2:**
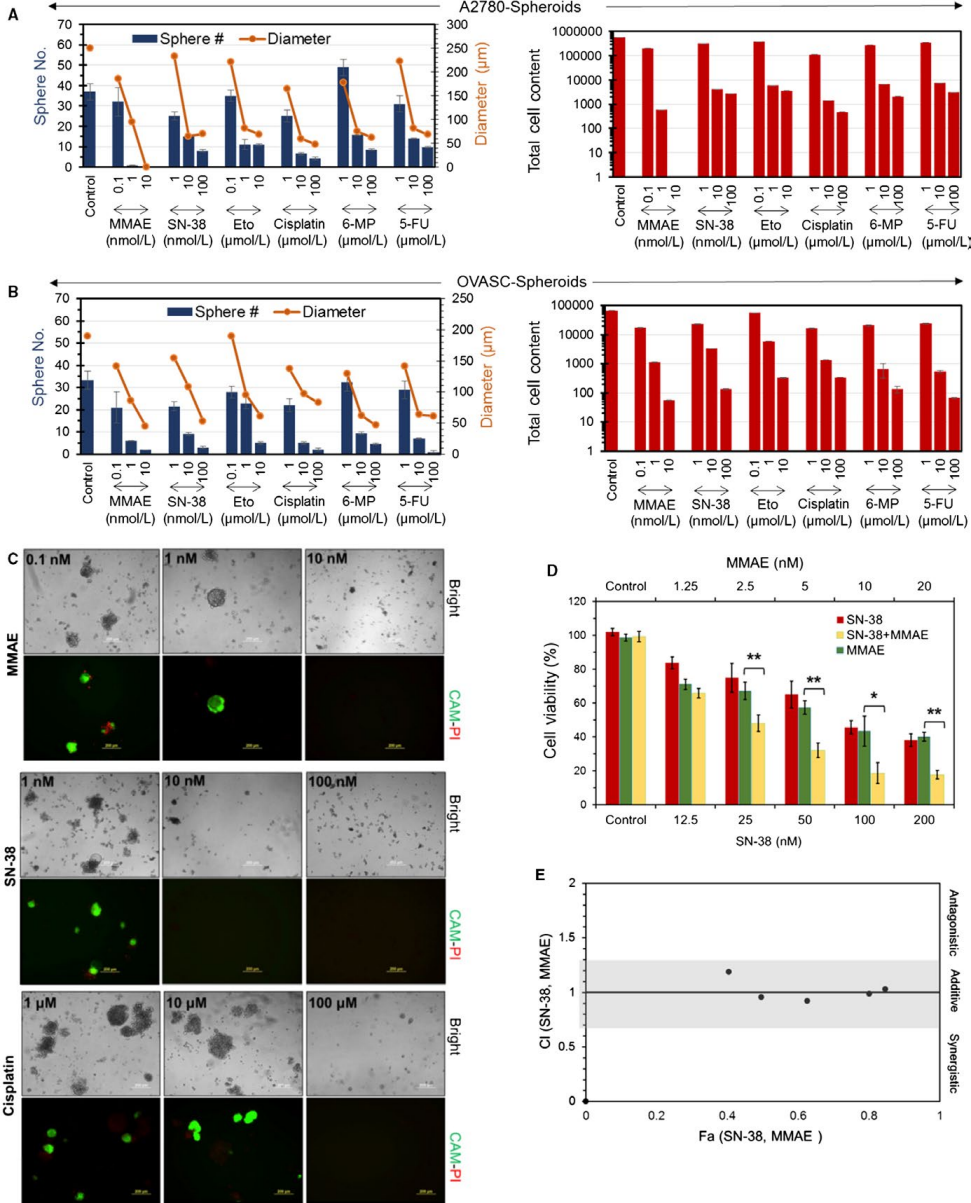
Evaluation of the effectiveness of the chemotherapeutic agents in killing CSC‐rich tumorspheres. A and B, Measurement of the sphere number, size and total cell content in tumorspheres after treatment with different chemotherapeutics. Data are presented as mean ± SD (n = 4). C, Evaluation of OVASC‐1 tumorsphere recurrence 30 d after treatment of primary tumorspheres with MMAE, SN‐38 and cisplatin. Tumorspheres were stained with the fluorophores; calcein‐AM (CAM) and propidium iodide (PI) to visualize live (green) and dead (red) cells respectively. Scale bar = 200 µm. D, Evaluation of the efficacy of single‐agent and combination therapy with MMAE and SN‐38 in killing OVASC‐1 cells (*t* test, *P < .05; **P < .01). Data are presented as mean ± SD (n = 4). E, Combination index plot for the determination of antagonistic, additive, or synergistic effects of SN‐38 and MMAE combination therapy. The additivity line is at CI = 1; CI < 1 indicates synergism and CI > 1 indicates antagonism. The additivity effect has some level of uncertainty, as depicted by the gray area 20

In the next experiment, we investigated the effectiveness of few chemotherapeutic drugs that have been used alone or as antibody‐drug conjugates in the past decades to treat ovarian cancer at different disease stages. Tested drugs included MMAE (microtubule‐disrupting agent),^12^, ^13^ SN‐38 (topoisomerase I inhibitor),^14^ etoposide (topoisomerase II inhibitor),^15^ cisplatin (DNA crosslinker),^16^ 6‐MP (nucleotide analogue and inhibitor of RNA and protein synthesis)^17^, ^18^ and 5‐FU (pyrimidine analogue and thymidine synthase inhibitor).^18^, ^19^ As we mentioned above, OVASC‐1 cells have been shown to be resistant to paclitaxel; therefore, we did not examine their sensitivity to this drug at the in vitro level to avoid redundancy. Both A2780 and OVASC‐1 tumorspheres were first generated and then treated with the aforementioned chemotherapeutic drugs at different concentrations. To examine the effectiveness of each drug, we first measured the change in tumorsphere number and size, and from those measurements we calculated the remaining total cell content in each tumorsphere after treatment. As expected, the results of this experiment showed a significant decrease in the number and diameter of tumorspheres in both cell lines with increasing drug dose (Figure 2A,B). We also observed that the effective dose that reduced the number of tumorspheres by 50% was in the nanomolar range for MMAE (1 nmol/L) and SN‐38 (10 nmol/L), whereas other drugs were effective in the micromolar range. To analyze the effectiveness of the drugs in a more meaningful way, we used a formula to estimate the total number of cells (total cell content) in each tumorsphere. In essence, the formula takes into account both tumorsphere diameter and number to calculate the total cell content. For example, knowing that the average diameter of an A2780 cell is 10 μm, the total number of cells in tumorspheres of the A2780 untreated control group was calculated to be 578 125 (N = 37) (Figure 2A). We observed that only MMAE was able to reduce the total cell content in A2780 tumorspheres to approximately 600 cells (<0.1%) at concentrations as low as 1 nmol/L. At a 10 nmol/L concentration, MMAE killed all of the cells in A2780 tumorspheres. By comparison, to achieve the same level of tumorsphere killing efficiency (ie <0.1%), a 100 μmol/L concentration of cisplatin had to be used. Although we observed the same trend in OVASC‐1 treated tumorspheres, it was apparent that the OVASC‐1 tumorspheres were far more resistant to therapy than the A2780 tumorspheres. For instance, to achieve the same level of tumorsphere killing efficiency (ie <0.1%), at least 10 nmol/L of MMAE was needed, which was ten times more than the needed amount for A2780 tumorspheres (Figure 2B).

Closer investigation of OVASC‐1 cells revealed that they exhibit overexpression of the MDR1 gene, which is responsible for mediating multidrug resistance.^5^ Therefore, it was very interesting to observe that the OVASC‐1 tumorspheres could be effectively killed with MMAE in such low concentrations, despite the findings of a recent report showing that this drug is a substrate for MDR1.^20^ The remarkable anticancer efficacy of MMAE could be attributed to its high potency, which allows this drug to kill cancer cells even at very low concentrations. The sensitivity of the OVASC‐1 cells to SN‐38 was also interesting. Some older studies have indicated that SN‐38 is a substrate for both the MDR1 and the ABCG2 drug efflux pumps,^21^ whereas recent evidence suggests that the ABCG2 pump is a key mediator of SN‐38 resistance.^22^, ^23^ To shed more light on this ambiguity, we measured the expression levels of the ABCG2 transporter in OVASC‐1 cells. The results of this experiment showed that more than 90% of OVASC‐1 cells expressed ABCG2. However, the expression level was in low intensity meaning that ABCG2 existed in low copy numbers on the cell surfaces (Figure S1). Since OVASC‐1 cells express MDR1 in high copy numbers but ABCG2 in low copy numbers, our data related to the sensitivity of OVASC‐1 to SN‐38 at nanomolar (nmol/L) concentrations appears to rule out MDR1 as a prominent mediator of SN‐38 resistance, and agree with the more recent studies reported in 2016.^22^, ^23^ This interesting observation highlights the importance of personalized therapy, in which an IP sample could be taken from each patient and analyzed for ABCG2 expression. If ABCG2 expression exists in low copy numbers, then administration of SN‐38 may be more effective than cisplatin, paclitaxel or a combination thereof.

So far, the in vitro data have shown that MMAE and SN‐38 have the greatest ability among the tested drugs to significantly reduce the cell content in CSC‐rich tumorspheres. This observation prompted us to examine the viability of the cells remaining inside the treated tumorspheres and evaluate their potential to regrow and regenerate the tumorspheres. For this purpose, we characterized the viability of the more resistant OVASC‐1 tumorspheres after treatment with one dose of either MMAE or SN‐38. Posttreatment, the OVASC‐1 tumorspheres were left undisturbed for 30 days in replenishing media to enable the growth of any remaining cancer cells that survived the therapy. After 30 days, the remaining tumorspheres were stained with calcein‐AM and propidium iodide to visualize the live/dead cells, respectively. The results of this experiment showed that both MMAE and SN‐38 could effectively inhibit tumorsphere regrowth at concentrations as low as 10 nmol/L (Figure 2C). This result indicates that at that concentration, all CSCs, as well as mature differentiated cancer cells were killed. In contrast, cisplatin was unable to completely eradicate CSCs even at 10 μmol/L concentration, as evidenced by the presence of live cells in the tumorspheres. When the concentration of cisplatin was increased to 100 μmol/L, the drug was able to completely kill the tumorspheres and inhibit relapse.

Thus far, the in vitro data highlight the high potency of MMAE and SN‐38 and their ability to kill CSC‐rich OVASC‐1 tumorspheres. However, this high potency could also result in significant toxicity when used in vivo. To investigate treatment options that might maintain high anticancer activity but reduce the potential for toxicity, we examined whether the combined use of MMAE and SN‐38 has an additive, synergistic or antagonistic effect. Therefore, OVASC‐1 cells were treated with combinations of MMAE and SN‐38 at a 1:10 ratio (based on equitoxic dose and IC_50_ values) and different concentrations. The CI was then calculated and plotted against the corresponding Fa. The results of this experiment demonstrated that MMAE and SN‐38 have an additive cytotoxic effect, making it possible to use both drugs together at lower concentrations without compromising anticancer activity (Figure 2D,E). This important observation led us to formulate our hypothesis for in vivo studies.

### 3.3 Evaluation of cancer progression, response to therapy, and recurrence in vivo

**Figure 3:**
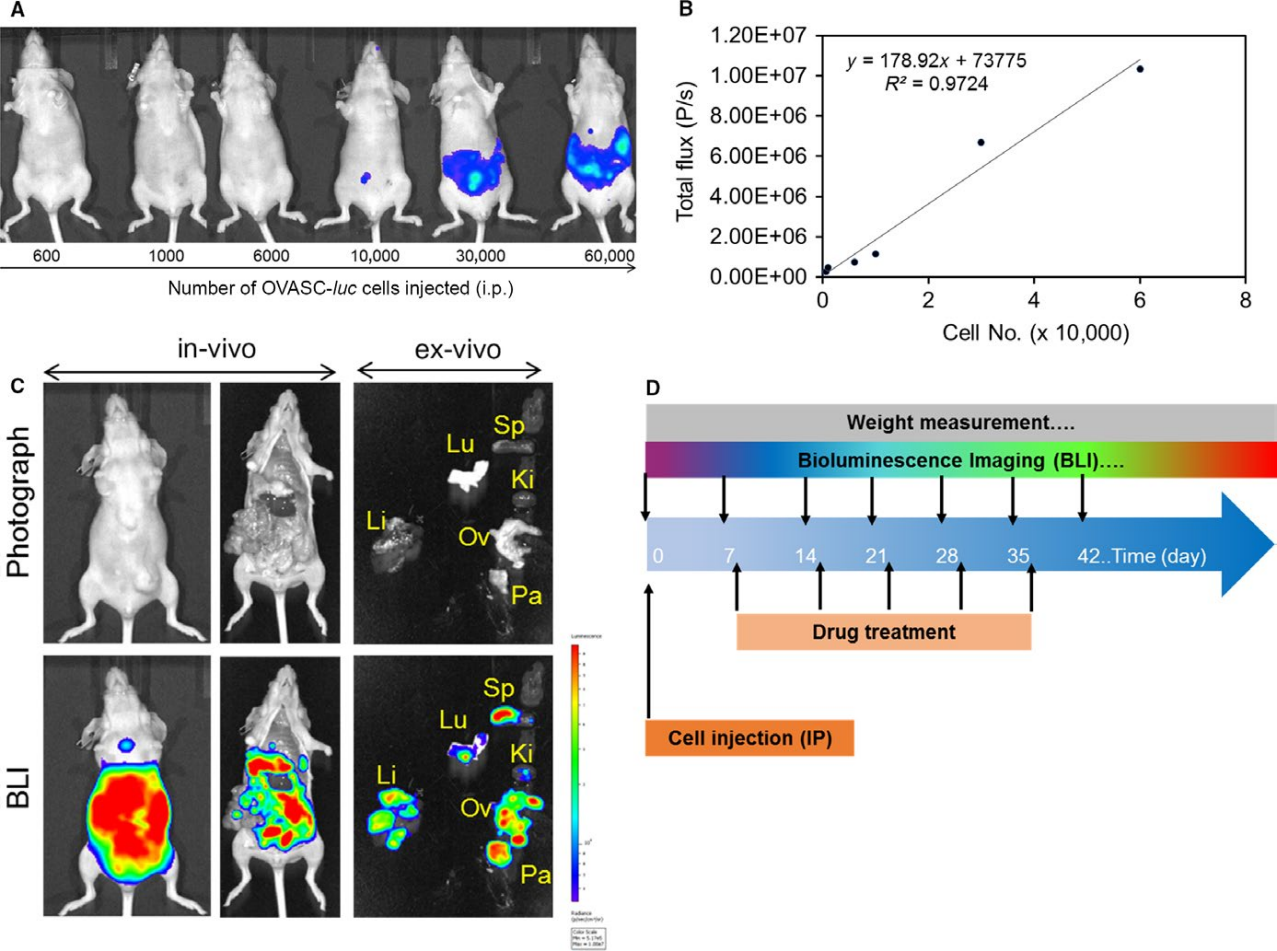
A, Bioluminescence of intraperitoneally injected OVASC‐*luc* cells in nude mice. B, Plot of cell number vs total flux and its correlation. C, Brightfield images (up) and BLIs (down) of whole body and individual organs 2 mo post intraperitoneal injection of five million OVASC‐*luc* cells. The BLI of mouse shows intact mouse as well as dissected organs such as liver (Li), lung (Lu), spleen (Sp), pancreas (Pa), kidney (Ki), ovary and associated reproductive organs (Ov). D, The drug dosing schedule, BLI schedule and weight measurement studies

To determine the biological relevance of these findings, we hypothesized that low dose therapy with MMAE and SN‐38 can not only eradicate the CSC‐rich IP tumorspheres but also inhibit relapse in mice. To test this hypothesis, we engineered luciferase expressing OVASC‐1 cells (OVASC‐*luc*) to allow us monitor disease progression over time in live nude mice. Before starting the treatment, we first measured the sensitivity of BLI to identify the minimum number of engineered OVASC‐*luc* cells that could be detected in mice after IP injections. The results of this experiment showed a linear correlation between the number of injected OVASC‐*luc* cells and the measured bioluminescence. The results also showed that the minimum number of cells that could be detected by the imaging system after IP injection was 10 000 with a total flux of 1.1 × 10^6^ (P/s) (Figure 3A,B). We also investigated whether the OVASC‐*luc* cells could survive the peritoneal environment in nude mice and metastasize to adjacent and distant organs, in a manner similar to its clinical course. Therefore, nude mice were injected with OVASC‐*luc* cells, and tumor growth was monitored over a 2 month period. The results of this experiment showed that OVASC‐*luc* cells thrived in the mouse abdomens, and quickly metastasized and formed tumor nodules in the lung, liver, pancreas, spleen and reproductive organs (eg ovaries) (Figure 3C). Based on these findings, we set up our in vivo studies, in which OVASC‐*luc* cells were injected into the peritoneum of mice and allowed to grow and stabilize for a week. The drug dosing schedule, BLI schedule and other parameters are shown in Figure 3D. In this pilot study, mice were randomly divided into nine groups (n = 3) and received treatments starting 8 days following tumor implantation (Table 1). Since the in vitro studies showed that the tumorspheres are fully formed by day 3, this lag period was provided to ensure their stable establishment.

**Table 1:** Drug doses and treatment response to various treatment regimens

Group no.	Drug name	Drug dose	Mice (n)	CR	PR	SR	NR	Recurrence
Group 1	Vehicle control	N/A	3				3	
Group 2	Cisplatin + paclitaxel	12 + 15 (mg/kg)	3		3			
Group 3	SN‐38	10 (μg/kg)	3		2		1	
Group 4	SN‐38	1 (mg/kg)	3	1	2			
Group 5	MMAE	1 (μg/kg)	3		2		1	
Group 6	MMAE	100 (μg/kg)	3	2	1			
Group 7	SN‐38 + MMAE	10 + 1 (μg/kg)	3		2	1		
Group 8	SN‐38 + MMAE	100 + 10 (μg/kg)	3	3				1 (2nd week)
Group 9	SN‐38 + MMAE	1000 + 100 (μg/kg)	3	3				0

CR: complete response meaning no evidence of the tumor. PR: partial response meaning decrease in tumor volume (≥50%). OR: overall response meaning CR+PR. SR: small response meaning decrease in tumor volume (≤25%); NR: no response meaning significant increase in tumor volume or appearance of new tumor(s). Recurrence: appearance of tumor after complete response.

**Figure 4:**
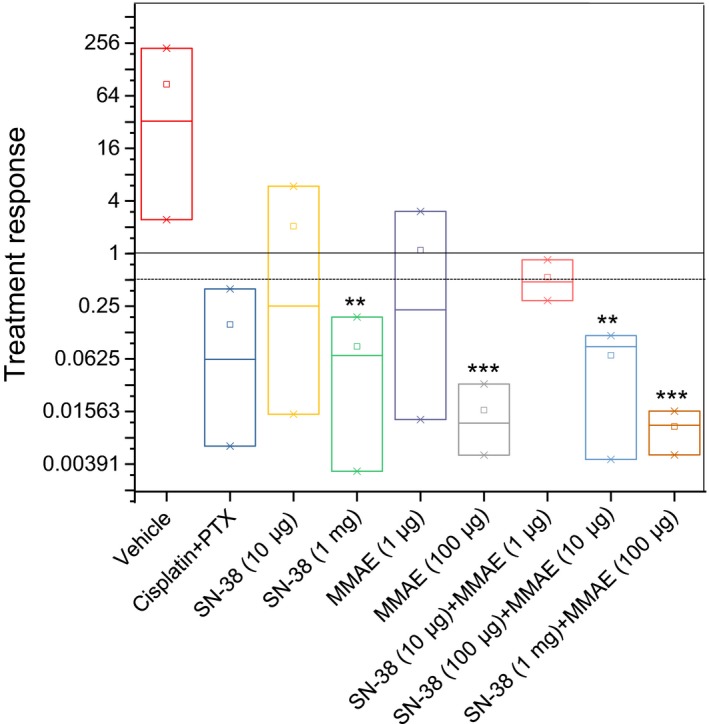
Box‐and‐whisker plot showing median (minimum‐maximum) and treatment response (total flux at the end of treatment/total flux before treatment). Data are presented as mean ± SD (n = 3). Statistics were computed by the paired *t* test (prior to vs. last treatment). *, **, and ***indicate *P*‐values <.05, <.01 and <.001, respectively

The untreated control group (Group 1) received vehicle solution only. We also assigned one control mouse group to be treated with cisplatin plus paclitaxel, which are standard‐of‐care drugs for recurrent ovarian cancer (Group 2). Mice in this group received the maximum tolerable dose of cisplatin (12 mg/kg) and paclitaxel (15 mg/kg), as reported previously.^24^ Two groups received ultra‐low and low doses of SN‐38 (Groups 3 and 4). Two groups received ultra‐low and low doses of MMAE (Groups 5 and 6). The final three groups received three different combinations of SN‐38 with MMAE (Groups 7‐9). The data related to each treatment group were analyzed to understand the disease progression and therapy response. The image analysis of mice in Group 1, which received vehicle solution (no drug), showed a steady‐state increase in the bioluminescence signal, indicating an increase in ascitic tumor mass over time (Figures 4 and S2). The mice in this group gained weight until they were euthanized due to an impediment of movement resulting from the increase in tumor size. The mice in Group 2, which received cisplatin plus paclitaxel, showed partial response to therapy (Figures 4 and S3, Table 1). The fold change in tumor mass as measured by BLI was 0.062 (0.039‐0.006) ([Median] [min‐max]) at the end of treatment compared to initial tumor mass prior to treatment (*t* test, *P *=* *.02). The mice in this group tolerated the treatment for at least 3 weeks but started to show severe signs of toxicity after the fourth dose and lost significant weight. Therefore, these mice were either euthanized when their body weight loss exceeded the 20% threshold, or their treatment was discontinued when other signs of toxicity were observed. Considering that the OVASC‐1 cells are samples from a patient with recurrent disease and are resistant to paclitaxel,^5^ this response rate was expected. Mice in groups 3, 5, and 7 that were treated with the ultra‐low concentration of SN‐38 (10 μg/kg) and/or MMAE (1 μg/kg) either as single agents or in combination did not show any response to therapy; the decrease in tumor mass was not statistically significant (*t* test, *P *>* *.05) (Figures 4 and S4‐S6, Table 1). All mice in these three groups gained weight and did not show any visible signs of toxicity.

**Figure 5:**
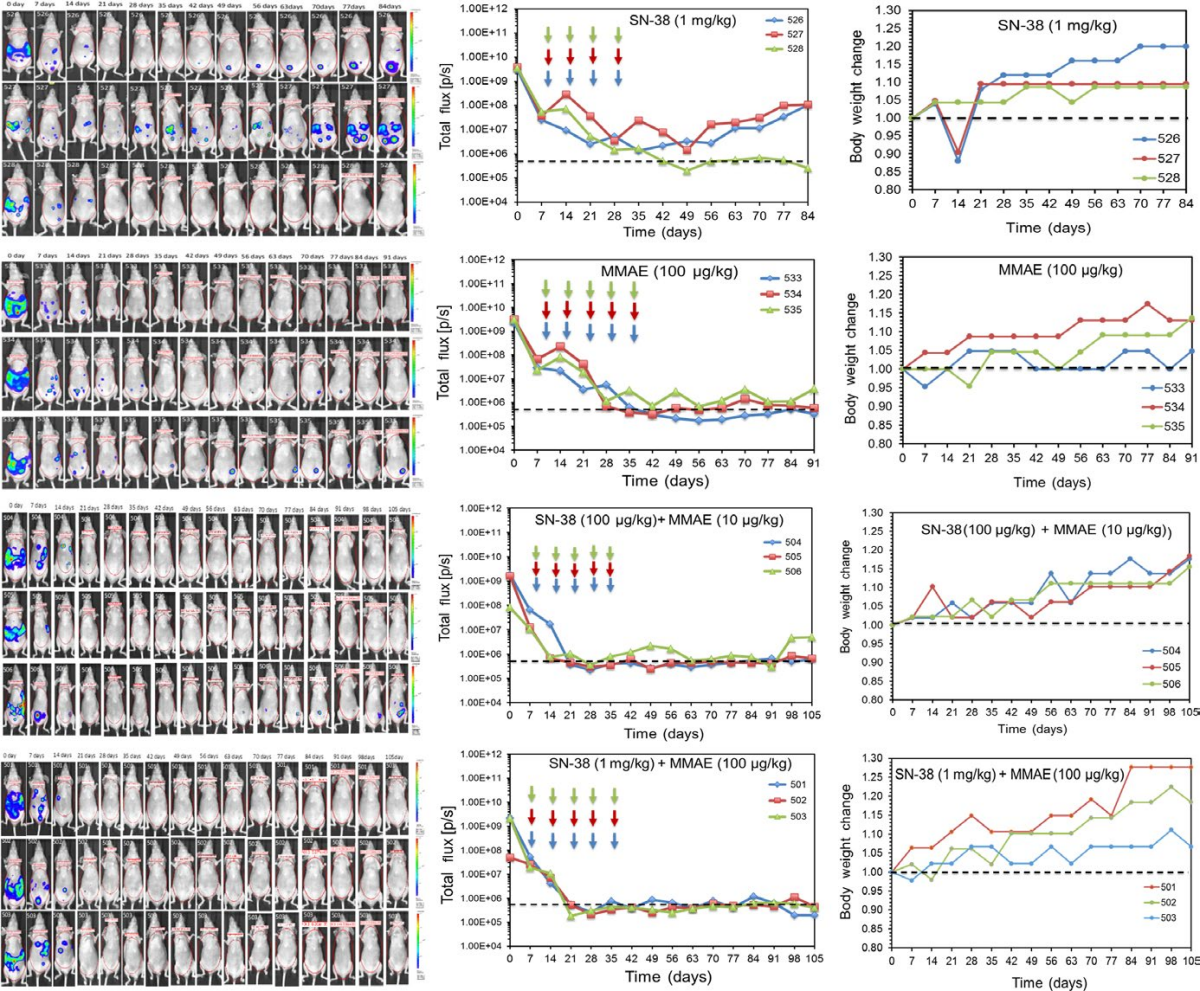
Evaluation of disease progression, recurrence and body weight change in the four treatment groups that responded to therapy. The dashed lines indicate the background mouse body bioluminescence which was determined to be 5 × 10^5^. Arrows indicate the days that mice received the drug treatments (ie days 8, 15, 22, 29, and 36)

Mice in Groups 4 and 6 that were treated with SN‐38 (1 mg/kg) or MMAE 100 μg/kg showed a statistically significant response to therapy (*t* test, *P *<* *.01) (Figure 4, Table 1). Notably, one mouse in each group completely responded to therapy and remained disease‐free for at least 3 months (Figure 5). All mice in these two groups gained weight and did not show any signs of visible toxicity. Mice in group 8, which were treated with a combination of SN‐38 (100 μg/kg) and MMAE (10 μg/kg), also showed a significant change in tumor mass (Figure 4, Table 1). All mice in this group showed a complete response and remained disease‐free without any sign of recurrence for at least 90 days (Figure 5). However, one mouse showed recurrence after 90 days. Additionally, mice in this group did not show any signs of toxicity. Finally, all mice in Group 9, which were treated with a combination of SN‐38 (1 mg/kg) and MMAE (100 μg/kg), showed a complete response to therapy (Figure 4, Table 1). Remarkably, mice in this group showed a complete response after administration of only four doses of the drug combination. Most importantly, no sign of cancer relapse was observed indicating the complete eradication of both differentiating cancer cells and CSCs; we could not detect a bioluminescence signal even after 105 days (Figure 5). Overall, four groups showed a statistically significant response to therapy. These groups included the mice that were treated with SN‐38 (1 mg/kg), MMAE (100 μg/kg), SN‐38 (100 μg/kg) plus MMAE (10 μg/kg), and SN‐38 (1 mg/kg) plus MMAE (100 μg/kg). However, only mice that were treated with a combination of SN‐38 (1 mg/kg) and MMAE (100 μg/kg) did not show any signs of recurrence. These data support our hypothesis that low‐dose therapy with MMAE and SN‐38 can not only eradicate the CSC‐rich IP tumorspheres but also inhibit relapse in mice.

It is worth noting that the number of animals per group in this proof‐of‐principle study was set at three to examine merely the effectiveness of IP low‐dose combination therapy with the two highly cytotoxic drugs (ie MMAE and SN‐38). The exciting outcome of this study encourages further investigation of this approach using larger number of animals.

### 3.4 Evaluation of tissue toxicity

**Figure 6:**
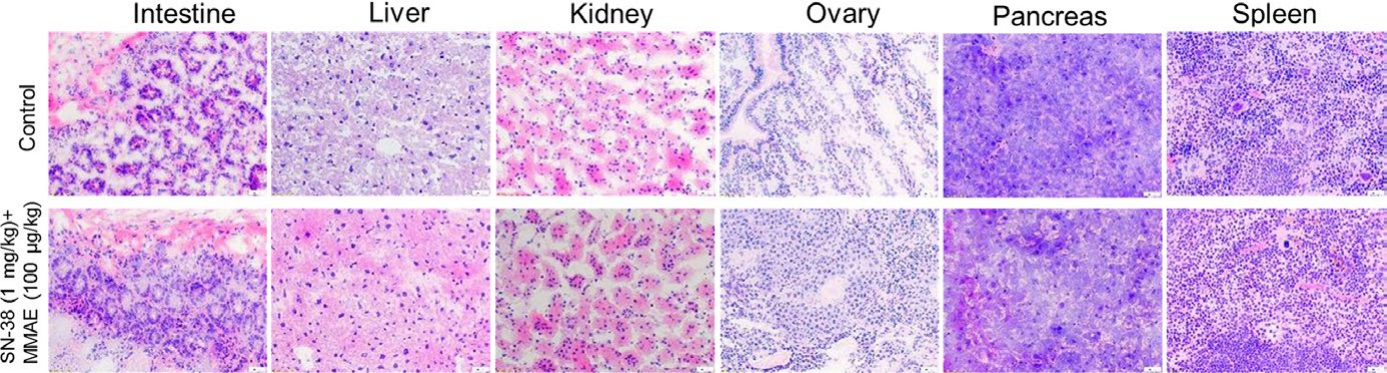
Hematoxylin and eosin (H& E) staining of dissected mouse organs. Cryosectioning was performed using a cryostat, followed by fixation and H&E staining. Photomicrography was conducted using a Leica microscope with a 20× objective

MMAE and SN‐38 are both highly potent anticancer drugs that are too toxic for use in an untargeted setting. As a result, they are mainly used as antibody‐drug conjugates (ADCs) for the treatment of variety of gynecologic malignancies including ovarian cancer.^25^, ^26^ In fact, for most ADCs currently in clinical development, dose‐limiting toxicities appear to be more closely associated with the anticancer drug and not related to the targeted antigen. Since the localized IP therapy with low‐dose SN‐38 (1 mg/kg) and MMAE (100 μg/kg) was effective and produced no visible signs of toxicity, we used histopathological methods on abdominal organs to investigate whether any toxicity occurred at the cellular level. The histopathological results did not reveal any notable toxicity to the abdominal tissues (Figure 6). In addition to evaluating histopathology, we monitored the mice in the MMAE‐treated groups for signs of peripheral neuropathy, including extreme sensitivity to touch, lack of coordination and falling, muscle weakness or paralysis, and bowel/bladder problems. Overall, all mice that were treated with either ultra‐low or low‐dose MMAE did not show any signs of peripheral neuropathy, which is a common MMAE‐associated toxicity.^27^ These findings indicate that the proposed regimen is not only effective but also tolerable to mice. The absence of serious toxicity to normal tissues could be attributed to the low‐dose, localized administration of the drugs.

## 4 CONCLUSIONS

One of the most common causes of death in patients with primary or recurrent ovarian cancer is metastasis to the peritoneal cavity. The standard therapy for metastatic ovarian cancer at this stage is cytoreductive surgery (CRS) of macroscopic disease, followed by IV and IP administration of anticancer drugs such as cisplatin or carboplatin in combination with paclitaxel. The addition of IP chemotherapy to a regimen of CRS and IV chemotherapy could delay cancer relapse in patients with very small residual tumors following surgery. However, IP chemotherapy has not been routinely used, mainly due to increased toxicity and potential complications. Our data show that IP administration of low‐dose MMAE and SN‐38 could effectively eliminate CSC‐rich ascites from the peritoneal cavity without inducing any significant toxicity. While MMAE and SN‐38 are not administered as free drugs due to their high potency and potential for systemic toxicity, our low‐dose localized therapy approach effectively restricted the cytotoxic effects to the tumor cells in the peritoneum. Consequently, maximum efficacy with minimal adverse effects was achieved. Considering that the OVASC‐1 cells showed resistance to therapy with cisplatin and paclitaxel but responded remarkably to combination therapy with MMAE and SN‐38, this study could represent a new approach for treating metastatic ovarian cancer without the need for IV chemotherapy. Further investigation into this approach could facilitate translation of this therapeutic regimen into the clinic either as first‐line therapy or in cases of acquired resistance to cisplatin and paclitaxel.

## CONFLICT OF INTEREST

None declared.
