# Supplementary material for: A novel chemotherapeutic protocol for peritoneal metastasis and inhibition of relapse in drug resistant ovarian cancer
Source: Cancer Med. 2018 Jun 21;7(8):3630–41. doi: 10.1002/cam4.1631 (PMC6089146; doi:10.1002/cam4.1631)
Supplement: Supplementary file 1 [file CAM4-7-3630-s001.docx]

**Supplementary Data**


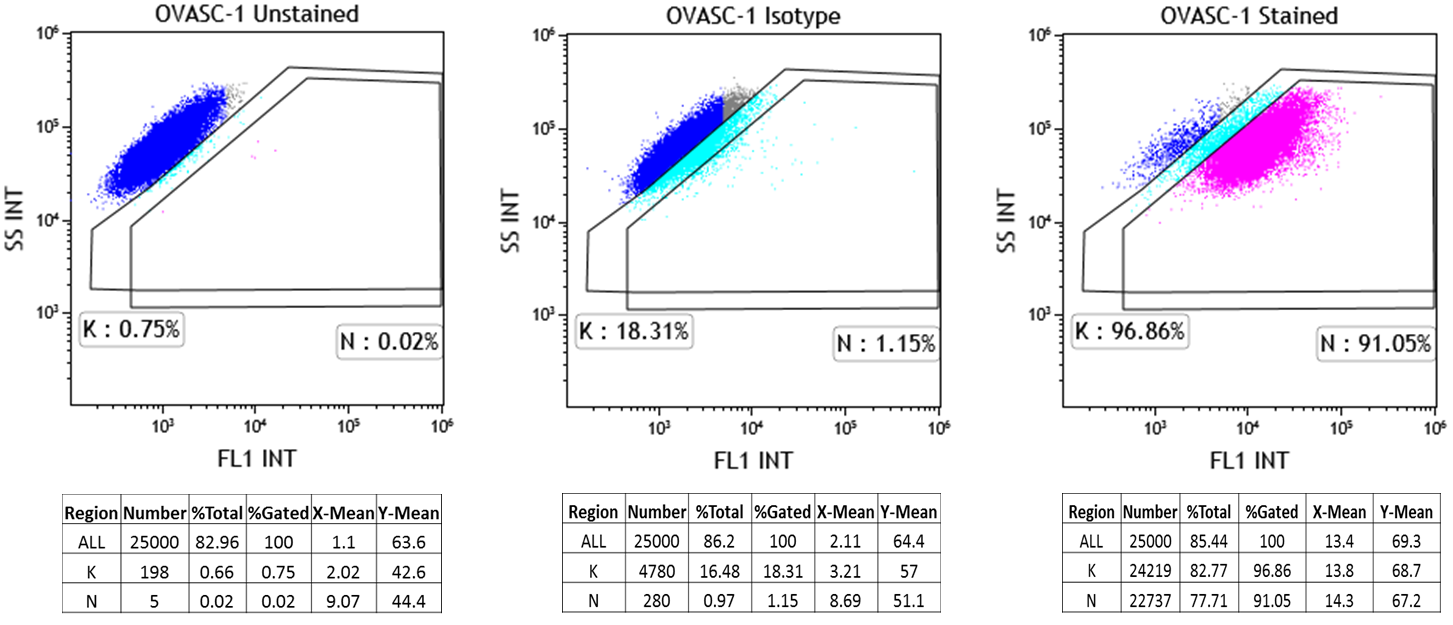


**Supplementary Figure S1:** OVASC-1 cells were fixed with 4% paraformaldehyde (10 min) followed by permeablization with 0.1% Tween/PBS (20 min). Cells were then incubated with Rabbit Anti-*h*ABCG2 primary antibody (Abcam) overnight at 4 ^o^C, and then stained with AlexaFluor conjugated secondary Goat Anti-Rabbit IgG antibody (room temperature, 1 h). The isotype was Rabbit IgG monoclonal antibody (Abcam). Data acquisition was carried out using Beckman Coulter Gallios Flow-cytometer and analysis was performed using Kaluza 1.5 module.

**
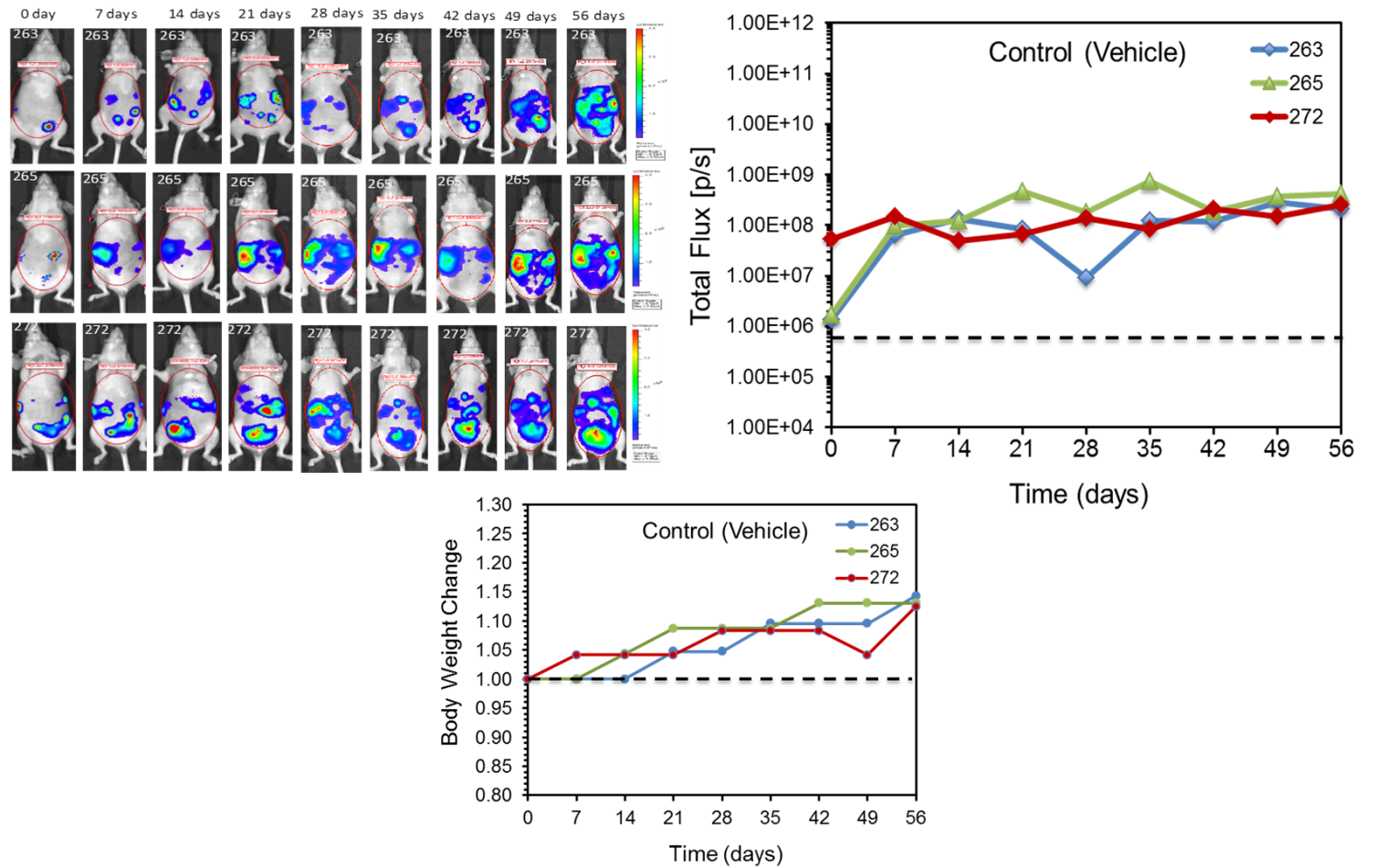
**

**Supplementary Figure S2:** Bioluminescence imaging and body weight change measurement of mice in control group treated with vehicle.

**
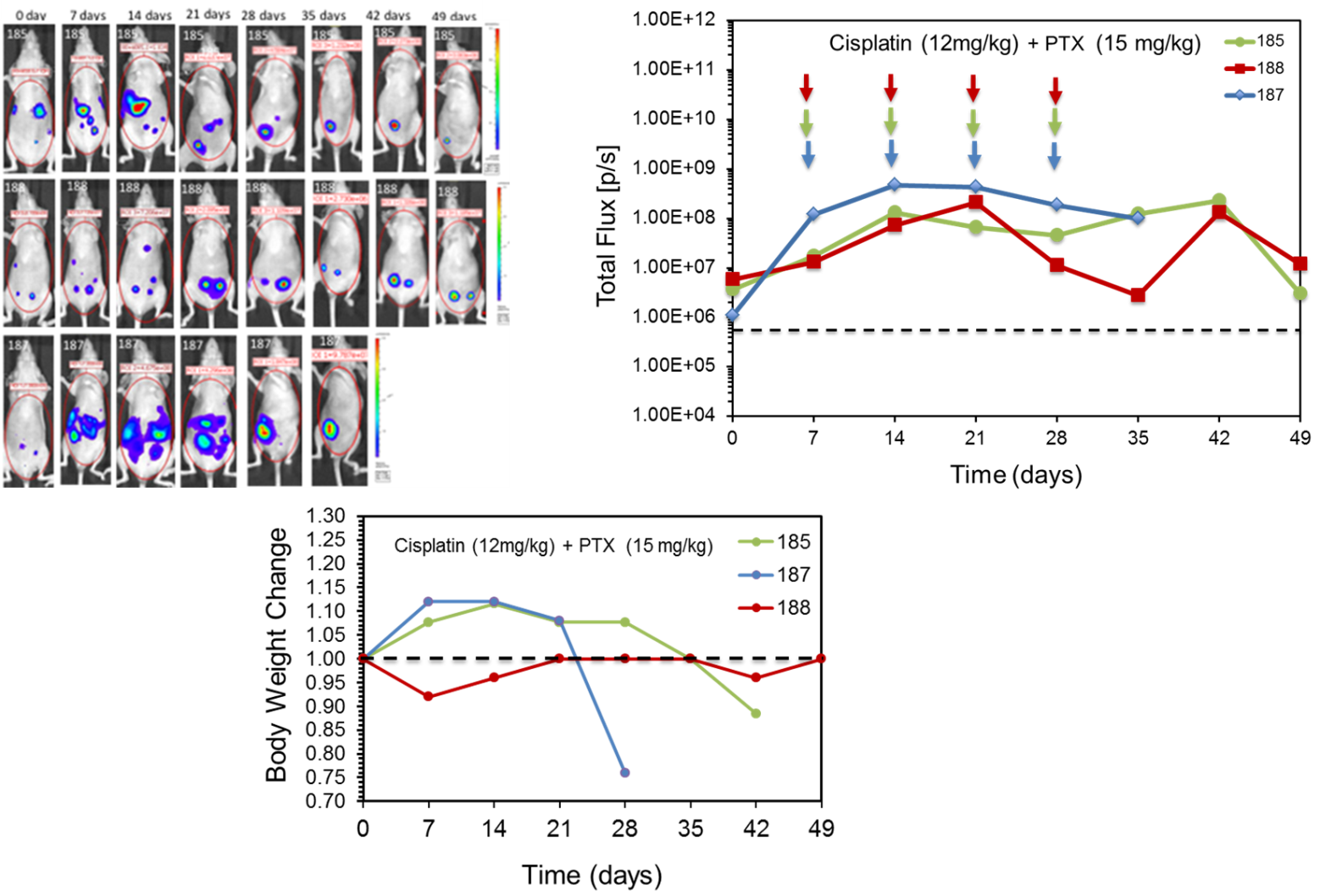
**

**Supplementary Figure S3:** Bioluminescence imaging and body weight change measurement of mice in group treated with cisplatin and paclitaxel.

**
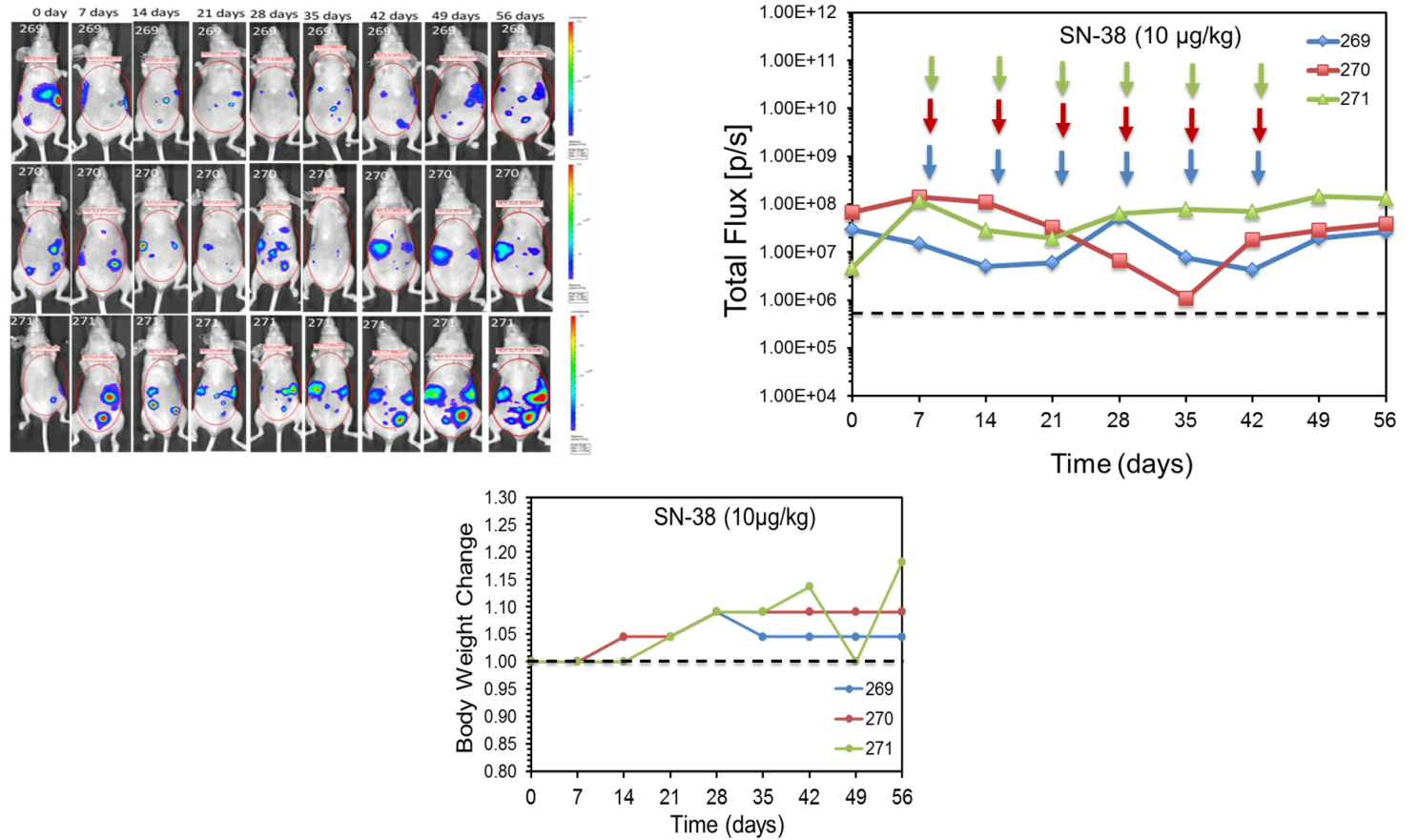
**

**Supplementary Figure S4:** Bioluminescence imaging and body weight change measurement of mice in group treated with SN-38 (10 µg/Kg).

**
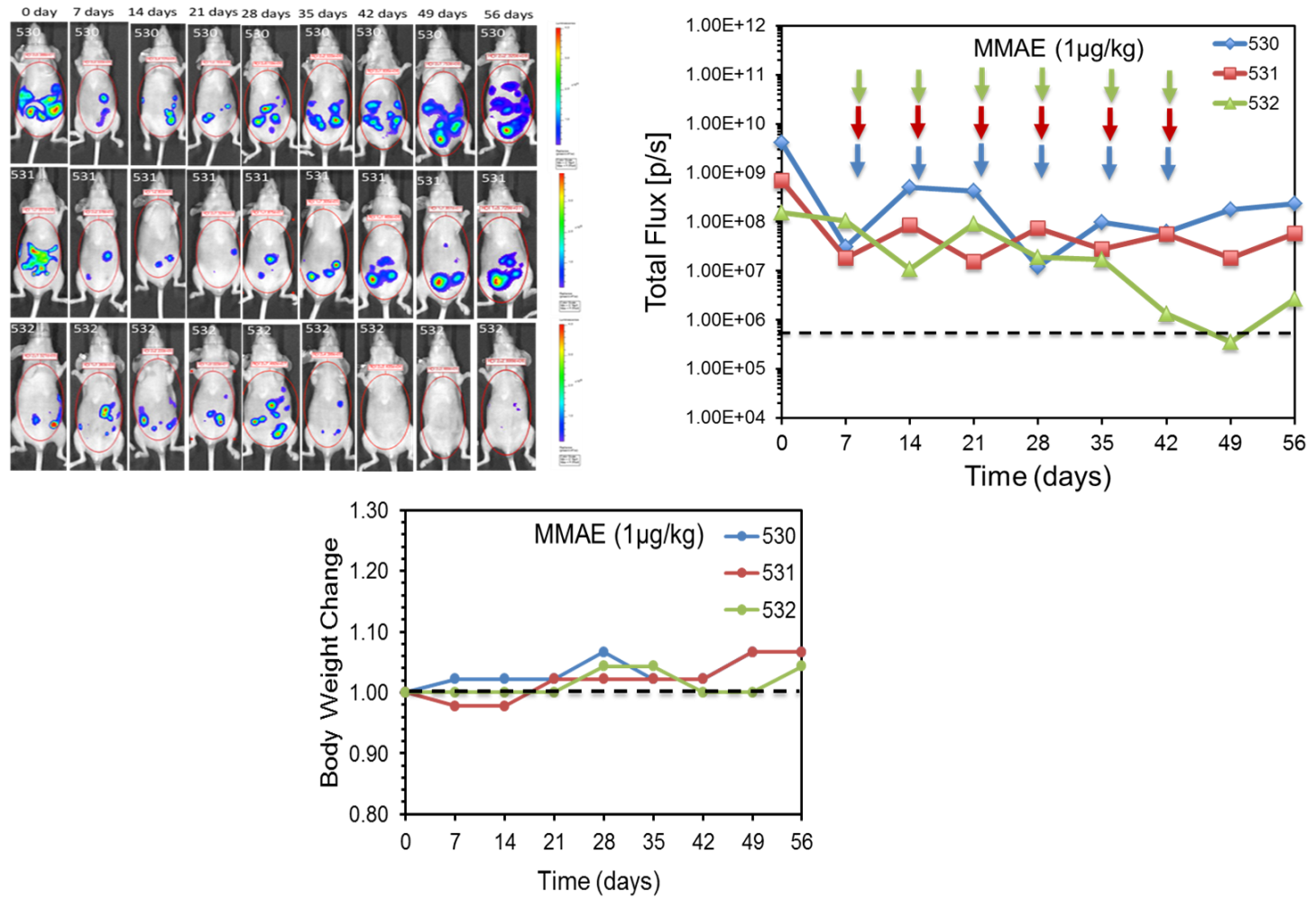
**

**Supplementary Figure S5:** Bioluminescence imaging and body weight change measurement of mice in group treated with MMAE (1 µg/Kg).

**
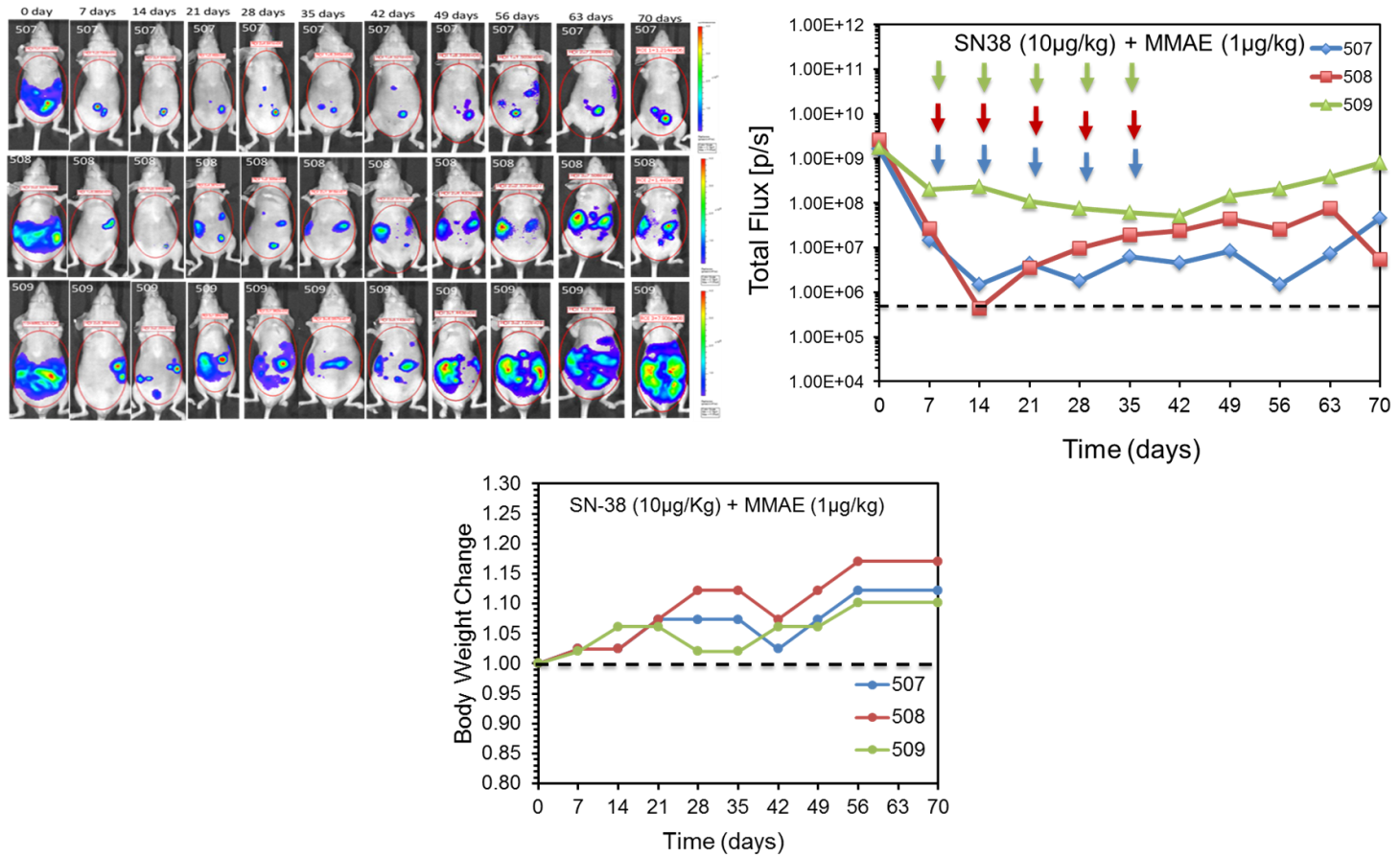
**

**Supplementary Figure S6:** Bioluminescence imaging and body weight change measurement of mice in group treated with SN-38 (10 µg/Kg) and MMAE (1 µg/Kg).

**Supplementary Table S1:** Tumorsphere proliferation kinetics and analysis. Parent population is represented by Generation-1 (G1), G-max (Generation with maximum % cells), Proliferation index (PI), Non-Proliferating Fraction (NPF), and Precursor Frequency (Pγ).

| **Cell line** | **Time (day)** | **G1 (%)** | **Gmax** | **PI** | **NPF** | **Pγ** |
| --- | --- | --- | --- | --- | --- | --- |
| **A-2780** | 0 | 93 | G1 | 1.04 | 0.96 | 0.001 |
|  | 1 | 51.69 | G1, G2 | 1.33 | 0.69 | 0.004 |
|  | 3 | 1.01 | G4 | 6.37 | 0.06 | 0.821 |
|  | 9 | 0.07 | G6 | 15.06 | 0.01 | 0.945 |
| **OVASC-1** | 0 | 99.81 | G1 | 1 | 1 | 0.0003 |
|  | 1 | 58.48 | G1 | 1.26 | 0.74 | 0.0004 |
|  | 3 | 7.86 | G3 | 2.7 | 0.21 | 0.411 |
|  | 9 | 0.93 | G5 | 8.78 | 0.08 | 0.83 |
